# Admission criteria and management of critical care patients in a pandemic context: position of the Ethics Commission of the French Intensive Care Society, update of April 2021

**DOI:** 10.1186/s13613-021-00855-z

**Published:** 2021-04-26

**Authors:** Olivier Lesieur, Jean Pierre Quenot, Zoé Cohen-Solal, Raphaëlle David, Laure De Saint Blanquat, Maxime Elbaz, Bénédicte Gaillard Le Roux, Cyril Goulenok, Sylvain Lavoué, Virginie Lemiale, Emmanuelle Mercier, Chaouki Mezher, Benoît Misset, Gwendolyn Penven, Anne Laure Poujol, Bertrand Quentin, Régis Quéré, Thierry Van Der Linden, Jean Philippe Rigaud

**Affiliations:** 1 Intensive Care Unit, General Hospital, La Rochelle, France; 2 Intensive Care Unit, University Hospital, Dijon, France; 3 Saint Louis University Hospital, Paris, France; 4 Paris Fire Fighters Brigade (BSPP), Paris, France; 5 Pediatric Intensive Care Unit, Necker University Hospital, Paris, France; 6 Clinical Sleep and Vigilance Center, Hôtel Dieu University Hospital, Paris, France; 7 Pediatric Intensive Care Unit, University Hospital, Nantes, France; 8 Intensive Care Unit, J. Cartier Private Hospital, Massy, France; 9 Intensive Care Unit, University Hospital, Rennes, France; 10 Intensive Care Unit, Saint Louis University Hospital, Paris, France; 11 Intensive Care Unit, University Hospital, Tours, France; 12 Intensive Care Unit, General Hospital, Trévenans, France; 13 Intensive Care Unit, University Hospital, Liège, Belgium; 14 University Hospital, Reims, France; 15 Intensive Care Unit, Pitié-Salpêtrière University Hospital, Paris, France; 16 Gustave Eiffel University, Marne-la-Vallée, France; 17 Organ Procurement Organization, Necker University Hospital, Paris, France; 18 Intensive Care Unit, Catholic University Hospital, Lille, France; 19 Intensive Care Unit, General Hospital, Dieppe, France

## Abstract

Intensive care unit professionals have experience in critical care and its proportionality, collegial decision-making, withholding or withdrawal of treatment deemed futile, and communication with patients’ relatives. These elements rely on ethical values from which we must not deviate in a pandemic situation. The recommendations made by the Ethics Commission of the French Intensive Care Society reflect an approach of responsibility and solidarity towards our citizens regarding the potential impact of a pandemic on critical care resources in France, with the fundamental requirement of respect for human dignity and equal access to health care for all.

Intensive care unit (ICU) professionals have experience in critical care and its proportionality, collegial decision-making, withholding or withdrawal of treatment deemed futile, and communication with patients’ relatives [1, 2]. These elements rely on ethical values from which we must not deviate in a pandemic situation.

The recommendations made below reflect an approach of responsibility and solidarity towards our citizens regarding the potential impact of a pandemic on critical care resources in France, with the fundamental requirement of respect for human dignity and fairness.

1. Age cannot be used as the sole criterion for admission nor denial of access to ICU settings. Careful assessment of the patient's medical characteristics (including age, comorbidities, previous autonomy and healthy life span) and the expected benefit of critical care remain the essential criteria for patient referral.
2. The decision-making procedures commonly used in the ICU to determine whether a patient is eligible for critical care apply to the pandemic situation. As far as possible, the medical decision not to admit a patient to the ICU must remain collegial and take into account the patient's wishes: first-person expression, advance directives, information from the appointed surrogate, relatives and attending physician(s). In this situation, it is essential to ensure that the care and accompaniment of the patient and his/her relatives is carried out in the most suitable environment and under the conditions usually recommended.
3. A patient's wish not to receive intensive care (first-person expression, advance directives, testimony from the surrogate, relatives or the attending physician) must be respected as long as it appears appropriate to the situation.
4. It would be inappropriate to encourage vulnerable persons or their legal representatives to draft advance directives (which in this case would not be) urgently on the pretext of imminent peril and scarcity of resources.
5. A patient should not be admitted to the ICU solely for the motive of infection with the pandemic’s causative agent if admission would otherwise have been declined due to an obvious unfavorable risk/benefit ratio (comorbidities, frailty, exposure to iatrogenic risk, prognosis).
6. The management of a patient not infected with the pandemic’s causative agent must follow the same principles as in a non-epidemic situation. A situation of health crisis should not lead to questionable choices influenced by the concomitant massive surge of infected patients (i.e., denying access to critical care to an uninfected patient who would otherwise have been admitted). Each patient must be considered in his/her individuality, whatever his/her pathology, and be treated fairly.
7. In cases of uncertainty about the prognosis, a time-limited trial of intensive care treatment—when it is feasible—allows to search for the patient's previously expressed wishes, to check the patient's history and comorbidities that were not identified at admission, and to assess, after a reasonable period of time, the evolution under maximal treatment [3]. At the end of this so-called "full code" period, it may be decided either to continue intensive care, or to withhold or withdraw therapies deemed disproportionate, and/or to refer the patient to a unit more suitable to his/her situation.
8. While in the ICU, the level of therapeutic involvement should be reassessed daily with the same thoroughness for patients infected with the pandemic’s causative agent as for other patients. The proportionality of the care provided to a patient suffering from serious comorbidities and presenting in a life-threatening situation must be evaluated collegially with the patient himself/herself when possible, his/her relatives (including the surrogate), his/her attending physician(s) and possibly a geriatrician in the case of an elderly person.
9. Healthcare institutions involved in the outbreak must have downstream structures (acute medicine, post-acute rehabilitation, mobile team and/or palliative care unit) that can immediately care for patients who no longer require life-sustaining treatments, and who need to be transferred to free up an ICU bed, although this is not the primary intention.
10. In case of non-admission to the ICU, palliative care facilities and mobile teams must be available 24 h a day to care for patients whose condition would be considered too serious and without hope of improvement through conventional treatment or intensive care, including in nursing homes or at home.
11. A debriefing with a psychologist must be proposed to the health care teams in charge of pandemic’s victims because of the intense emotional charge induced by this exceptional situation. An identical competency must be specifically dedicated to the patients' relatives, especially since they are kept away by the containment measures.
12. ICU beds made available in regions less impacted by the epidemic must be used and patients transferred to sites guaranteeing the best possible care, in agreement with the physicians in charge of these units.
13. When the available resources become scarce, their distribution will take into account patients' comorbidities and previous autonomy, evolution under treatment in case of a so-called "trial of intensive care treatment" and individual chances of survival with an acceptable quality of life.
14. The drafting of prioritization algorithms—necessity making the law—by healthcare professionals must: (1) be primarily based on the ethical background, knowledge, skills and expertise of our specialty; (2) plan graduated or alternative strategies (delayed admission, inter-hospital transfer) in case of saturation of healthcare structures in the territories concerned; (3) take into account in their formulation the risk of political, legal and media manipulation of the documents produced during the crisis when it comes to an end.
15. The protection of health care teams in contact with patients infected with the pandemic’s causative agent cannot be sacrificed on the grounds of rationing or depletion of available resources. Securing healthcare facilities is an imperative prerequisite for the optimal management of patients infected or not infected by the pandemic’s causative agent.
16. All medical decisions and resulting care modalities must be documented in patient records, including those declined in the ICU.
17. It is crucial to take into account the prohibition of relatives’ visits and to provide quality distant communication facilities (i.e., phone, video-conference). Access must be organized for the cult representatives when required.

## Data Availability

Not applicable.

## Abbreviation

ICU: Intensive care medicine
